# Efficacy of imidacloprid 10%/moxidectin 1% spot-on formulation (Advocate®) in the prevention and treatment of feline aelurostrongylosis

**DOI:** 10.1186/s13071-020-3937-2

**Published:** 2020-02-12

**Authors:** Lea Heuer, Gabriele Petry, Matthias Pollmeier, Roland Schaper, Katrin Deuster, Holger Schmidt, Katrin Blazejak, Christina Strube, Angela Di Cesare, Donato Traversa, Manuela Schnyder, Janina McKay-Demeler, Georg von Samson-Himmelstjerna, Sandra Mangold-Gehring, Claudia Böhm

**Affiliations:** 1 Bayer Animal Health GmbH, Leverkusen, Germany; 2 BioMedVet Research GmbH, Walsrode, Germany; 3 Institute for Parasitology, Centre for Infection Medicine, University of Veterinary Medicine Hannover, Hannover, Germany; 4 Faculty of Veterinary Medicine, University of Teramo, Teramo, Italy; 5 Institute of Parasitology, University of Zürich, Zürich, Switzerland; 6 Institute of Parasitology and Tropical Veterinary Medicine, Free University of Berlin, Berlin, Germany; 7 Dawbuts Pty Ltd, Camden, NSW Australia

**Keywords:** *Aelurostrongylus abstrusus*, Moxidectin, Feline lungworm, Prevention, Treatment

## Abstract

**Background:**

In three randomized, controlled laboratory efficacy studies, the efficacy in the prevention of patent infections of a topical combination of imidacloprid 10%/moxidectin 1% (Advocate® spot-on formulation for cats, Bayer Animal Health GmbH) against larval stages and immature adults of *Aelurostrongylus abstrusus*, as well as the treatment efficacy of a single or three monthly treatments against adult *A. abstrusus*, were evaluated.

**Methods:**

Cats were experimentally inoculated with 300–800 third-stage larvae (L3). Each group comprised 8 animals and the treatment dose was 10 mg/kg bodyweight (bw) imidacloprid and 1 mg/kg bw moxidectin in each study. Prevention of the establishment of patent infections was evaluated by two treatments at a monthly interval at three different time points before and after challenge infection. Curative efficacy was tested by one or three treatments after the onset of patency. Worm counts at necropsy were used for efficacy calculations.

**Results:**

In Study 1, the control group had a geometric mean (GM) of 28.8 adult nematodes and the single treatment group had a GM of 3.4 (efficacy 88.3%). In Study 2, the control group had a GM of 14.3, the prevention group had a GM of 0 (efficacy 100%), while the treatment group had a GM of 0.1 (efficacy 99.4%). In Study 3, the GM worm burden in the control group was 32.6 compared to 0 in all three prevention groups (efficacy 100% for all of those groups).

**Conclusions:**

The monthly administration of Advocate® reliably eliminated early larval stages and thereby prevented lung damage from and patent infections with *A. abstrusus* in cats. Regarding treatment, a single application of Advocate® reduced the worm burden, but it did not sufficiently clear the infection. In contrast, three monthly treatments were safe and highly efficacious against *A. abstrusus*.
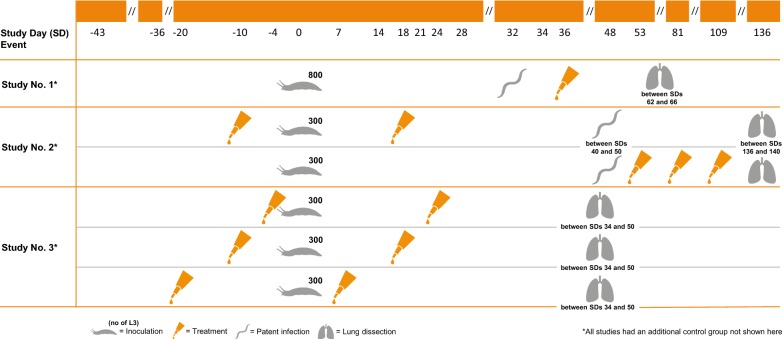

## Background

Parasitic respiratory diseases are of increasing importance in feline clinical practice [1]. Over recent years, the number of reports of feline lungworm infections has risen worldwide, particularly across mainland Europe. Concerning parasitic helminths of the feline respiratory tract, such as *Oslerus rostratus*, *Troglostrongylus* spp. or *Capillaria aerophila*, *Aelurostrongylus abstrusus* remains the most important of the feline lungworms in both clinical and epidemiological terms [1, 2]. This nematode is distributed worldwide with varying prevalence rates in cats, e.g. 1.7% in the UK [3], 5.1% in the USA [4], approximately 8% in Denmark [5] or Greece [6], and between 10 and 38.3% in Italy [7]. The parasite may infect domestic cats and occasionally wild felids, e.g. European wildcats or lions [2, 8, 9].

Generally, the risk of infection is higher for stray or free-roaming cats in endemic areas [10–12]. Infections may be acquired by ingestion of either the intermediate hosts (mollusks) or paratenic hosts, such as birds, rodents, amphibians or reptiles [13–16]. Besides the adult nematodes, which reside mainly in the bronchioles, alveolar ducts and lung parenchyma, egg production and migrating larvae can also cause inflammatory lung lesions, depending on the infective dosage [17]. Infections can range from subclinical or severe to fatal. Clinical manifestations include non-specific signs compatible with a systemic disease, such as apathy, depression and weight loss, as well as respiratory alterations, e.g. dyspnea, panting, coughing, sneezing and nasal discharge [15, 18, 19]. Concerning bronchoscopy findings, bronchiectasis and mucus accumulation were found in cats infected with *A. abstrusus* [20]. Cardiac presentations, such as right-sided cardiomegaly, heart murmurs and pulmonary hypertension, have also been described in kittens [21]. Furthermore, spontaneous death during anesthesia has also been associated with lungworm disease in cats [22]. Severe cardiopulmonary disease or fatalities can be the result of a late diagnosis and treatment of aelurostrongylosis [1].

Ideally, infections should be prevented or treated at an early stage with adequate actives. Routine deworming is therefore advisable, especially in free-roaming cats with respiratory signs. Data on the efficacy of treatments are available from clinical studies and case reports, but information on preventive properties of the licensed products is limited. Use of an oral formulation of milbemycin oxime and praziquantel (Milbemax®, Elanco Europe, Bad Homburg, Germany) stopped larval shedding and resolved clinical signs in a single case after two applications two weeks apart [21]. Selamectin 45 mg spot-on formulation (Stronghold®, Zoetis, Berlin, Germany; dose range 2.6–7.5 kg body weight, bw) was used twice 23 days apart in naturally infected cats and stopped larval shedding in 9 out of 10 cats [23]. Of the benzimidazoles, an oral paste with 18.75% fenbendazole (Panacur®; MSD Animal Health, Unterschleißheim, Germany) is licensed in the UK, among other countries, for the treatment of feline aelurostrongylosis. In two field studies [24, 25], an efficacy of 99.3% has been reported with the oral treatment of 50 mg/kg bw daily for 3 consecutive days. In these field studies, a single application of an emodepside/praziquantel (Profender®, Bayer Animal Health GmbH, Leverkusen, Germany) spot-on formulation at a dosage of 3 mg/kg emodepside and 12 mg/kg praziquantel showed a comparable efficacy, i.e. 99.4%, to the oral fenbendazole treatment in terms of larval shedding [25]. The emodepside formulation has been evaluated further in two randomized, placebo-controlled experimental studies. Two spot-on administrations at a two-week interval showed a 99.2% reduction of worm counts in the lung tissue [26]. Profender® is therefore licensed for the treatment of adult stages of *A. abstrusus* in cats with this treatment regimen [27]. Another product licensed for the treatment of *A. abstrusus* (L3, L4 and adults) is a topical formulation containing fipronil 8.3% w/v, (S)-methoprene 10% w/v, eprinomectin 0.4% w/v and praziquantel 8.3% w/v (Broadline®, Merial, Hallbergmoos, Germany), which had an efficacy of 99.6% against adult *A. abstrusus* [28, 29]. Knaus et al. [28] also demonstrated an efficacy with this formulation of 98.9%, 99.3% and 91.6% against L3, L4 and immature adults of *A. abstrusus*, respectively, showing the preventive potential against patent *A. abstrusus* infections.

Because of its longer half-life and its good safety profile, moxidectin has been suggested as another potential option for the chemoprevention of aelurostrongylosis. This drug remains detectable in plasma samples for weeks and consistent monthly administrations of topical moxidectin can induce elevated and sustained steady-state plasma concentrations in dogs and cats [30–32]. Furthermore, moxidectin has proven to be highly effective against patent *A. abstrusus* infections: in a field study, a spot-on formulation containing 10% imidacloprid and 1% moxidectin (Advocate®; Bayer Animal Health GmbH) showed 100% efficacy in reducing larval shedding and resolving clinical signs after a single treatment [24]. This formulation (Advocate®, Bayer Animal Health GmbH) is licensed in some markets (e.g. New Zealand, Australia) for the treatment and control of *A. abstrusus* in cats [33, 34].

The controlled laboratory studies reported here were conducted to investigate the efficacy of this formulation in the treatment and prevention of patency in cats experimentally infected with *A. abstrusus*.

## Methods

### Study design

**Fig. 1:**
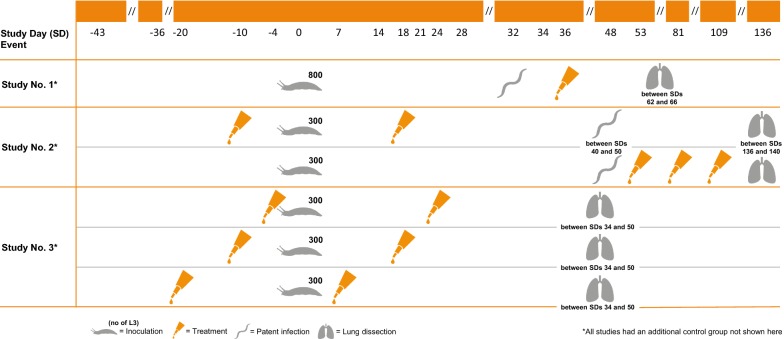
Overview of the study designs of the three experimental laboratory studies

To evaluate imidacloprid 10%/moxidectin 1% spot-on formulation for the prevention (against L3 and L4 larvae) and treatment (adults) of patent experimental *A. abstrusus* infections in cats, three studies were conducted. An overview is given in Fig. 1 and Table 1.

**Table 1:** Overview on study designs of the three laboratory studies

Study	Inoculation dose (L3)	Inoculation (study day)	Study group	Treatment	Study day	Necropsy (study day)
1	800	0	1.1	Placebo	36	62–66
			1.2	Advocate®	36	
2	300	0	2.1	Untreated control		136–140
			2.2	Advocate®	− 10; 18	
			2.3	Advocate®	53; 81; 109	
3	300	0	3.1	Placebo	− 4; 24	48–50 (20 cats)
			3.2	Advocate®	− 4; 24	34–35 (12 cats)
			3.3	Advocate®	− 10; 18	48–50
			3.4	Advocate®	− 20; 8	48–50

Studies were conducted in accordance with VICH Guideline 9, “Good Clinical Practice” (July 2001) [35], VICH Guideline 7, “Efficacy of anthelmintics: general requirements” [36] and VICH Guideline 20, “Efficacy of anthelmintics: specific recommendations for felines” [37]. The study procedures adhered to the WAAVP guidelines for evaluating the efficacy of anthelmintics in dogs and cats [38]. The study design was based on the scientific knowledge and experience gained in a similar study [26], because the guidelines do not include specific information on *A. abstrusus*.

#### Study 1

Study 1 evaluated the efficacy of a single treatment against *A. abstrusus* infections in cats after the onset of patency. The study was performed as a placebo-controlled, randomized and blinded laboratory study.

#### Study 2

In Study 2, the treatment and the prevention effect of imidacloprid 10%/moxidectin 1% spot-on formulation were examined. In the prevention group, the cats were treated before and after inoculation. In the second group, the treatment efficacy was examined by applying the spot-on formulation after the onset of patency and twice thereafter at monthly intervals. The control group was not treated and blinding was only applied to necropsy procedures

#### Study 3

Study 3 evaluated the ability of imidacloprid 10%/moxidectin 1% spot-on formulation to prevent the patent infection when administered monthly for two consecutive months, starting before the cats were experimentally infected with *A. abstrusus* L3. The study was performed as a placebo-controlled, randomized and blinded laboratory study.

### Study animals

The cats used in all three studies were purpose-bred and either owned by the study facility or purchased from commercial breeders. The sex of the included animals was distributed across the studies as follows: 8 females/8 males in Study 1; 17 females/7 males in Study 2; 16 females/16 males in Study 3.

All cats were acclimatized at least 7 days before study inclusion. On the day of infection, the animals were aged less than 10 months. They had been vaccinated against major feline infectious diseases and they were dewormed once with pyrantel embonate (Banminth®, Study 1) or with pyrantel embonate and praziquantel (Drontal®, Studies 2 and 3) before the start of the study. Fecal samples were examined during the acclimatization period to verify successful deworming. Cats included in the studies were not allowed to have been recently treated with drugs (e.g. macrocyclic lactones) which could interfere with the investigational product.

Animals were group-housed in their respective study groups, but were housed individually on the day of treatment, for two consecutive days and for individual fecal sample collection. Pens were equipped with planks and boxes. Toys and scratching places were offered for environmental enrichment. The animals received standard commercial wet and/or dry food and water was available *ad libitum*. Handling and housing were performed in accordance with national animal welfare regulations.

### Allocation and treatment

All treatments in the three studies were administered with the minimum treatment dose of 10 mg/kg BW imidacloprid and 1 mg/kg BW moxidectin, corresponding to 0.1 ml of the spot-on formulation of Advocate® per kg BW.

In Study 1, the cats were randomly allocated to one of two study groups, with each group containing eight animals. The cats were first separated by sex and then ranked in descending order based on their highest fecal L1 count on study day (SD) 2 and 4. After randomization, each study group consisted of four male and four female cats. Treatment was administered once on SD 0, 36 days post-infection (dpi), once all cats had shown a positive fecal larval count. The cats in the treatment group received one dose of the recommended minimum treatment doses, while cats in the control group received a placebo formulation at a dose of 0.1 ml/kg bw.

In Study 2, 24 cats were randomly allocated to one of three study groups of 8 animals each, based on sex and body weight. To evaluate the prevention efficacy, treatment of the cats assigned to group 2 was administered twice on SD 10 (10 days prior to infection) and on SD 18 (18 dpi) (see Table 1). To evaluate the treatment efficacy, the cats in group 3 were treated three times at monthly intervals on SD 53, SD 81 and on SD 109. The cats assigned to the control group (group 1) were left untreated.

In Study 3, 32 cats were randomly allocated to one of four study groups, based on their sex and ranking according to body weight and animal ID. After randomization, each group consisted of 8 cats with equal sex distribution. The cats in the three treatment groups received a dose of the minimum treatment dose, whereas the cats in the control group were treated with a placebo formulation. The preventive efficacy of the imidacloprid 10%/moxidectin 1% spot-on formulation in the elimination of early larval stages (L3 and/or L4) of *A. abstrusus* was evaluated. Therefore, the cats assigned to group 1 (control) and group 2 were treated on SD 4 and on SD 24, group 3 was treated on SD 10 and SD 18 and group 4 on SD 20 and on SD 8.

### Health observations

All cats were handled and observed by the animal caretakers for their general health once daily from the beginning of the respective study to the day of necropsy for each individual. The animals were physically examined at least once before infection, before treatment and before necropsy. Only healthy cats were included in the studies. On treatment days, the cats were clinically assessed for adverse events at least twice at the start of treatment and up to 5 h afterwards.

### Experimental infections

The cats were experimentally inoculated before treatment with 800 (group 1.1 and 1.2) or 300 (group 2.3), or after treatment with 300 (groups 2.2 and 3.2, 3.3, 3.4) infective L3 of *A. abstrusus* (see Table 1). Due to animal welfare reasons, the number of larvae used for inoculation was reduced from 800 (Study 1) to 300 (Studies 2 and 3). The L3 were obtained by digestion of the experimentally infected snails. The snails had been experimentally infected with L1 which were obtained from naturally infected cats. The snails were cut into pieces and the snail tissue was minced and then digested for 20–30 min in HCl solution with pepsin at approximately 41 °C: 0.4–0.6 g pepsin was mixed in 100 ml Aqua Bidest and 0.7 ml of 37% HCl solution. The digested material was passed through a 180 µm sieve and centrifuged at 500×*g* for 5 min before the supernatant was discarded. A number of subsamples were counted under a stereo microscope. Individual inoculation doses for the cats were prepared and applied intragastrically *via* a stomach tube. The cats were anesthetized before inoculation with a combination of medetomidine (0.008 mg/kg bw) and ketamine (10 mg/kg bw) with or without premedication with acepromazine (0.15 mg/kg bw). In order to prevent vomiting or regurgitation, the cats received metoclopramide (0.3 mg/kg bw) intramuscularly. The cats were observed for vomiting or regurgitation for 60 min after inoculation. During these first 60 min, vomiting occurred in some cats (seven cats in Study 1, three cats in Study 3), but the amount of vomit was small (< 1 ml). In Study 3, two of the three cats were re-inoculated with 150 and 120 L3 based on the amount of vomit and larval counts in the vomit. In Study 1, the cats which had vomited were not re-inoculated, but as two cats showed spillage during inoculation, they were re-dosed with approximately 150 L3. In Study 2, none of the cats showed any vomiting within the first 60 min of inoculation. Between 2 and 3 h after inoculation, the majority of cats vomited; no re-inoculation was performed.

### Fecal examination

The feces of all cats had to be negative for nematode eggs and larvae before study inclusion and inoculation with *A. abstrusus*. To demonstrate the negative parasitological status of the individual cat, at least one fecal sample was examined using a combined sedimentation/flotation method and the Baermann technique during the acclimatization period. In Study 1, L1 counts were determined by a slightly modified quantitative Baermann technique. An exact amount of 4–10 g was used, 14 ml of fluid was taken from the sediment of the funnel and was centrifuged at 500×*g* for 2 min. The supernatant was discarded and, if the number of larvae was low, the whole sediment was screened, or if the number of larvae was high, an aliquot was screened for larvae and the total number calculated. The determined larval counts were additionally used for the randomization of cats.

Post-treatment fecal examinations were conducted as follows: in Study 1, starting at 30 dpi, pooled fecal samples per cage, i.e. two animals, were examined using the Baermann technique and once the fecal samples were positive for L1, individual fecal samples were collected and examined. In Study 2, individual fecal samples were collected and examined every other day between SD 40 and 48 (study groups 2.1, 2.3) or rather SD 50 (study group 2.2) to detect the start of patency. In Study 3, post-treatment fecal examinations were conducted on the day of necropsy.

### Necropsy

In the three studies, necropsy was performed on several consecutive study days, depending on the days of treatment (details in Table 1). The lungs and heart of each cat were completely removed. All lung lobes were cut into pieces of approximately 1 cm^3^ and pieces were divided into two approximately equal portions. Lung tissue was checked for lungworms under a stereomicroscope by dissecting each piece with scalpel and forceps. The two portions of each lung piece were analyzed by at least two different persons in order to randomize the potential differences in the dissection technique. Lungworms were identified according to species, observed for viability and counted. If larval or pre-adult stages were present, this was also recorded and, if necessary, verified under an optical microscope. As worms can embed themselves very deeply into the lung parenchyma, it had to be considered that originally viable worms could have been destroyed and cut into pieces during isolation from lung tissue. Worms could have also died because of the long duration of the complicated worm counting procedure. To evaluate viability and the onset of death, each lungworm, or part of a worm, was carefully examined under a stereomicroscope. Viability/motility, general appearance and structure of the integument were assessed. All viable worms and all recently-dead intact worms or worm pieces with intact integument were counted as viable worms. Specimens of *A. abstrusus* which were obviously dead before necropsy started (i.e. lytic worms with marked changes of the cuticle) were counted as dead worms. In the case of worm parts, these were differentiated between nematode heads and/or tails. Each present head and/or tail was counted. If the number of heads was greater than the number of present tails, the heads were used to calculate the total number of worms. If the number of tails was greater, the tails were used for this calculation. Total worm counts determined in necropsy are listed in Additional file 1.

### Efficacy and statistical analysis

As per specification in VICH Guidelines 7 and 20 [33, 34], the adequacy of infection was achieved when at least six cats in the control group were confirmed to be infected and when the lower 95% confidence intervals of the geometric means were greater than 10% of the geometric means.

Percent efficacy was calculated according to the recommendations for controlled tests described in the abovementioned guidelines:

$$\% {\text{ Effectiveness }}\left( {\text{reduction}} \right) \, = \, \left( {{\text{N2 }}{-}{\text{ N1}}} \right)/{\text{N2}} \times 100$$

where N1 is the geometric mean worm count for the treatment group and N2 is the geometric mean worm count for the control group.

The geometric mean could not be calculated if the worm count values equaled zero. Due to this fact, a translation of all values was performed by adding 1 to the number of worm counts prior to logarithmic transformation and 1 was subtracted from the antilog value to meaningfully represent the geometric mean for each group. In Study 1, efficacy calculations were based on total worm counts (number of animals with ≥ 1 worm and geometric mean worm counts per sex and per group). The data distribution was considered normal and a parametric analysis of variance was used to test for a treatment group effect. In Study 2, the primary efficacy parameter to evaluate the preventive efficacy of imidacloprid 10%/moxidectin 1% spot-on formulation against L3/L4 of *A. abstrusus*, as well as the treatment efficacy against adult worms, was the number of viable adult worms at necropsy. Because the data were not normally distributed, the Wilcoxon-Mann-Whitney test was used to test for a treatment group effect.

In Study 3, the viable *A. abstrusus* worm counts were also not normally distributed. For this reason, the non-parametric Wilcoxon rank-sum test (two-tailed; alpha = 0.05) was used to test for treatment group effects.

In Studies 1 and 3, another lungworm, *Troglostrongylus brevior*, was found during necropsy. *Troglostrongylus brevior* L1 seems morphologically very similar to *A. abstrusus* L1. Therefore, the fecal larval results at necropsy were used for informational purposes only and no further statistical analysis was performed: the fecal larval counts had to be estimated as combined *A. abstrusus* and *T. brevior* L1 counts and no valid data could be generated for the reduction of fecal *A. abstrusus* L1 excretion.

All analyses were performed using SAS version 9.2 or 9.3 (SAS Institute, Cary, North Carolina, USA), respectively.

## Results

### Fecal examinations

Fecal examinations demonstrated L1 shedding in all cats in the untreated control groups and in the treatment group of Study 2 (before treatment) between SD 40 and SD 50, thus confirming patency and successful experimental *A. abstrusus* infections. All treatment and prevention groups had negative fecal larval counts at necropsy.

### Efficacy evaluation

In all three studies, the requirements for adequate infection were met, as all control cats were infected and harbored the required minimum number of parasites. The results are summarized in Table 2.

**Table 2:** Efficacy of imidacloprid 10%/moxidectin 1% spot-on in prevention and treatment of *Aelurostrongylus abstrusus* infection as determined in three controlled studies

Study	Group	Cats with ≥ 1 worm	Total^a^/viable^b^ worm burden^c^	Efficacy (%)	Adequacy of infection
1	(1) Control	8	28.8 (11–68)^a^	na	Yes**
	(2) Advocate® (SD 36)	7	3.4* (0–18)^a^	88.3	na
2	(1) Control	8	14.3 (4–42)^b^	na	Yes**
	(2) Advocate® SD 10 + SD 18	0	0*^b^	100	na
	(3) Advocate® SD 53, 81, 109	1	0.1* (0–1)^b^	99.4	na
3	(1) Control	8	32.6 (3–86)^b^	na	Yes**
	(2) Advocate® SD 4 + SD 24	0	0*^b^	100	na
	(3) Advocate® SD 10 + SD 18	0	0*^b^	100	na
	(4) Advocate® SD 20 + SD 8	0	0*^b^	100	na

^a^Efficacy calculation based on total worm counts

^b^Efficacy calculation based on viable worm counts

^c^Geometric mean (range)

*Significantly different (*P *< 0.05) compared to control group

**Lower bound of 95% confidence interval greater than 10% of the geometric mean

*Abbreviation*: na, not applicable

#### Study 1

At necropsy, control cats (group 1.1) harbored 6–39 viable worms and a total of 11–68 (viable + non-viable) adult *A. abstrusus* in their lungs (GM: 28.8). The total worm counts in the treated group 1.2 (single treatment imidacloprid 10%/moxidectin 1% spot-on formulation at 36 dpi) were reduced by 88.3% (t-test: *t*_(21) _= 4.70, *P *= 0.0001).

#### Study 2

In the control group (group 2.1), 4–42 viable worms (GM: 14.3) and a total of 4–44 worms (viable and non-viable) (GM: 14.4) were discovered at necropsy. The preventive group 2.2 (treatment with imidacloprid 10%/moxidectin 1% spot-on formulation 10 days before and 18 days after infection) did not harbor any nematodes at necropsy, resulting in a preventive efficacy of 100%. In the treatment group 2.3 (treatment after patency was reached with imidacloprid 10%/moxidectin 1% spot-on formulation on days 53, 81 and 109 post-infection), one animal harbored one viable worm at necropsy (efficacy 99.4%). In both of these groups, reductions were significant compared to the untreated control group (Wilcoxon rank sums test: *Z* = 3.5366 and 3.4562, respectively, *P *< 0.05).

#### Study 3

In Study 3, three different sets of pre- and post-infection preventative treatments, the latter then during pre-patency, with imidacloprid 10%/moxidectin 1% spot-on formulation at monthly intervals starting at 4, 10 or 20 days before infection were evaluated. The control group 3.1 harbored 3–86 viable worms (GM: 32.6) and a total of 3–88 worms (GM: 34.2). No viable worms were found at necropsy in any of the three prevention groups. One dead worm was found in one cat in group 3.3 (treatment on SD 10 and SD 18). Live L1 were found in the lungs of 6 out of 8 control cats at necropsy, while no live larvae were found in any of the treated cats. One cat in group 3.4 (treatment on SD 20 and SD 8) had one dead larva. All three imidacloprid 10%/moxidectin 1% treated groups showed a significant reduction in viable and total worm counts compared to the control group (Wilcoxon rank sums test: for viable counts, *Z *= − 3.5336 for all 3 pair-wise comparisons, *P *< 0.05, for total worm counts, *Z *= − 3.5366, − 3.4562 and − 3.5366, respectively, *P *< 0.05). Efficacy based on geometric mean viable worm counts for Advocate® was 100% in all three study groups.

### *Troglostrongylus brevior* infection

Unintended infections with *T. brevior* at necropsy (worm count range 2–23) were detected in six control cats in Study 1, as well as seven control cats (worm count range 1–4) and two cats (worm count range 1–2) in treatment groups 3.2 and 3.4, respectively, in Study 3. The numbers of detected *T. brevior* were not used for efficacy calculations. In Study 2, no coinfections with *T. brevior* were observed at necropsy. As *T. brevior* shed L1, which are morphologically almost identical to those shed by *A. abstrusus* and no L1 differentiation was performed, larval numbers in feces potentially represent the larvae of both species.

### Safety evaluation and general health

**Fig. 2:**
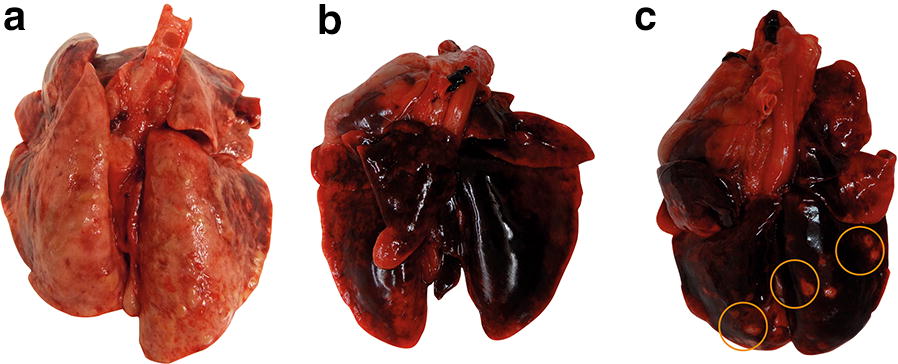
Lungs of *A. abstrusus*-infected cats at necropsy (Study 2). **a** Control group: verminous pneumonia, multifocal subpleural nodules, areas of consolidation, diffuse emphysema. **b** Prevention group: normal lung tissue, acute congestion. **c** Treatment group: multifocal subpleural nodules (orange circles), acute congestion

Treatment-related adverse events did not occur in any of the three studies. However, signs of lungworm infections such as tachypnea/dyspnea or sneezing, especially in the control groups, were frequently observed. This correlated with the severity of macroscopic findings at necropsy (Fig. 2). For example, in Study 2, all cats in the untreated control group showed macroscopic changes of the lung tissue: five out of eight lungs were described as “inhomogeneous” and four of these had a “meat-like” consistency. All of the eight lungs showed atelectasis to a varying extent. Three out of eight lungs showed nodules and the other five had vesicles on the surface. Even though the cats in the treatment group showed mostly normal lung tissue, all of them had white nodules varying in number and size and, in the lungs of one cat, atelectatic areas were observed. These changes are indicative of tissue damage caused by verminous pneumonia occurring before patency is reached.

In contrast, cats in the prevention group showed no or only small macroscopic changes to the lungs, i.e. the lung tissue of one cat was dark red (otherwise normal) and another had a few white nodules.

## Discussion

Reduction of the worm burden needs to be at least 90% in order to claim efficacy of an anthelmintic (VICH Guidelines 7 and 20 [36, 37]). A single treatment with the imidacloprid 10%/moxidectin 1% topical formulation at the recommended minimum treatment dose of 10 mg/kg bw imidacloprid and 1 mg/kg bw moxidectin resulted in 88.3% efficacy in Study 1. In contrast, a single application of the same dose in naturally infected cats, compared with a control oral formulation containing 18.75% fenbendazole (Panacur®, MSD), resulted in 100% reduction in fecal larval shedding [24]. However, as the duration of infection and the true parasite burden is unknown in naturally infected cats, a direct comparison to experimental infections is not feasible.

Likewise, three treatments in monthly intervals using the same imidacloprid 10%/moxidectin 1% formulation and dosage resulted in an efficacy of 99.4% in Study 2. This is concordant with other findings, i.e. that a complete reduction of larval shedding following a single treatment with a macrocyclic lactone is typically not achieved [23, 28], particularly when patent infections with high numbers of lungworms are present.

Efficacy in preventing such patent infections was assessed in Studies 2 and 3 and this is, to our knowledge, the first report of prevention of the establishment of a patent *A. abstrusus* infection with the use of a topical imidacloprid 10%/moxidectin 1% formulation at monthly intervals. This demonstrates its efficacy against larval and adult stages of *A. abstrusus*, as the treatment time points in Studies 2 and 3 were chosen based on the time points of the moults of *A. abstrusus* L3 (5–6 dpi) and L4 (8–9 dpi) [14]. Thus, *A. abstrusus* were expected to be L3 up to 4 dpi, L4 up to 7 dpi, L5 (immature adults) at around 14 dpi and mature adults from 25 dpi, the earliest time point at which worms have been reported to lay eggs in the lungs [39]. However, due to the known persistence of moxidectin in the treated animals, it cannot be clearly stated which of the larval stages were actually affected. The complete efficacy against larval and adult stages of *A. abstrusus* has previously only been described for a topical combination formulation containing the avermectin eprinomectin based on larval counts [28]. After a second treatment with this formulation at monthly intervals, efficacy remained > 99% up to the end of follow-up, but at necropsy, lungworm burdens were only reduced by 75% compared to controls [29]. This illustrates the shortcomings of efficacy calculations based on larval counts, as larval shedding may have stopped while adult worms persisted in the lung tissue [15], as also confirmed by the presence of specific antibodies against *A. abstrusus* in cats without larval excretion [40]. Furthermore, larval counts are influenced by irregular shedding and the limitations of the Baermann technique [15, 41, 42]. Because of these shortcomings, efficacy calculations in the studies presented here were to be based on adult worm counts in the lung tissue. Therefore, necropsies were performed to assess the worm burden in the lungs. Concerning the differing numbers of inoculated larvae in the three studies (800 L3 in Study 1, 300 L3 in Studies 2 and 3), it should be noted that the inoculation dosage of 800 larvae led to a variety of clinical and respiratory signs, as well as remarkably affected lung tissue. Due to animal welfare reasons, Studies 2 and 3 were conducted with a lower (300 L3) inoculation dose. However, despite the lower inoculation dose, the adequacy of infection was still met in both studies (see Table 2). In two of the studies presented here, it became evident at necropsy that some cats were infected with a second lungworm species, *T. brevior*. Such a co-infection can easily be overlooked when only larval counts are assessed, as the L1 of *A. abstrusus* and *T. brevior* are morphologically almost identical [43]. As no other differentiation between larvae was performed before the infection of snails (L1) and cats (L3), a partial infection with low numbers of *T. brevior* cannot be excluded. However, as numbers of identified *T. brevior* were considerably low, results were excluded from efficacy calculations. In contrast, adult specimens of *A. abstrusus* and *T. brevior* can be readily distinguished [26].

Prevention of patent infections by the use of anthelmintic drugs requires efficacy against early larval stages (L3 and/or L4) of *A. abstrusus*. This is highly desirable as verminous pneumonia with tissue damage and its clinical consequences may occur before the onset of patency [15, 17]; this is also evident from the macroscopic findings at necropsy (Fig. 2). Furthermore, preventive treatment can be a contribution to an effective epidemiological control of *A. abstrusus* infections in cats, since even after curative treatment, infected cats still shed L1 for up to 8.9 ± 2.0 days [44]. Because of its pharmacokinetic properties, such as a longer half-life and its favorable safety profile, moxidectin has been a promising candidate for the chemoprevention of an *A. abstrusus* infection [31, 45]. In other parasites, the chemopreventive properties of the moxidectin steady-state have already been shown, e.g. the formulation of imidacloprid 10%/moxidectin 1% protects cats against *Dirofilaria immitis* for 28 days after the last treatment when administered four times at monthly intervals [32]. Here, in two separate controlled laboratory studies, the chemoprevention of *A. abstrusus* infections with a topical imidacloprid 10%/moxidectin 1% formulation with 100% efficacy when administered twice at a monthly interval is shown.

## Conclusions

The monthly administration of Advocate® reliably eliminated early larval stages and thereby prevented lung damage from and a patent infection with *A. abstrusus* in cats. Regarding treatment, a single application of Advocate® reduced the worm burden, but it did not sufficiently clear the infection. In contrast, three monthly treatments were safe and highly efficacious against *A. abstrusus*.

## Supplementary information

**Additional file 1: Table S1.** *A. abstrusus* and *T. brevior* counts at necropsy (Study 1). **Table S2.** *A. abstrusus* counts at necropsy (Study 2). **Table S3.** *A. abstrusus* counts at necropsy (Study 3).

## Data Availability

All relevant data supporting the conclusions of this article are included within the article and its additional files.

## Abbreviations

BW: Body weight
dpi: Days post-infection
GM: Geometric mean
SD: Study day
