# Supplementary material for: Efficacy of imidacloprid 10%/moxidectin 1% spot-on formulation (Advocate®) in the prevention and treatment of feline aelurostrongylosis
Source: Parasit Vectors. 2020 Feb 12;13:65. doi: 10.1186/s13071-020-3937-2 (PMC7017503; doi:10.1186/s13071-020-3937-2)
Supplement: Supplementary file 1 — Additional file 1: Table S1. A. abstrusus and T. brevior counts at necropsy (Study 1). Table S2. A. abstrusus counts at necropsy (Study 2). Table S3. A. abstrusus counts at necropsy (Study 3). [file 13071_2020_3937_MOESM1_ESM.docx]

| **Additional file 1: Table S1.** *A. abstrusus*-and *T. brevior* counts at necropsy (study no. 1) | | | | | | | | |
| --- | --- | --- | --- | --- | --- | --- | --- | --- |
| **Study group**^1^ | **cat ID** | ***A. abstrusus*** | | | ***T. brevior*** | | | **living larvae present** |
|  |  | **viable** | **non viable** | **total** | **viable** | **non viable** | **total** |  |
| 1 | 8750 | 19 | 0 | 19 | 8 | 0 | 8 | yes |
|  | 9445 | 6 | 21 | 27 | 2 | 0 | 2 | yes |
|  | 7907 | 6 | 38 | 44 | 1 | 4 | 5 | yes |
|  | 4809 | 7 | 4 | 11 | 0 | 0 | 0 | yes |
|  | 4765 | 11 | 2 | 13 | 0 | 0 | 0 | yes |
|  | 6666 | 39 | 29 | 68 | 16 | 7 | 23 | yes |
|  | 6652 | 7 | 33 | 40 | 14 | 1 | 15 | yes |
|  | 6681 | 32 | 19 | 51 | 7 | 0 | 7 | yes |
| 2 | 6670 | 0 | 2 | 2 | 0 | 0 | 0 | no |
|  | 4810 | 0 | 3 | 3 | 0 | 0 | 0 | no |
|  | 6737 | 0 | 1 | 1 | 0 | 0 | 0 | no |
|  | 2044 | 3 | 0 | 3 | 0 | 0 | 0 | no |
|  | 7055 | 6 | 5 | 11 | 0 | 0 | 0 | yes |
|  | 6679 | 5 | 13 | 18 | 0 | 0 | 0 | yes |
|  | 6656 | 0 | 0 | 0 | 0 | 0 | 0 | no |
|  | 2733 | 1 | 4 | 5 | 0 | 0 | 0 | yes |
| ^1^ Treatment Group 1 = Placebo-treated control group SD 36  2 = Advocate® Spot-on at 0.1 mL/kg on SD 36 | | | | | | | | |

**Additional file 1: Table S2.** *A. abstrusus-*counts at necropsy (study no. 2)

| **Study group**^1^ | **cat ID** | **viable *A. abstrusus*** | | | | **non-viable *A. abstrusus*** | | | | **total worm counts**^2^ |
| --- | --- | --- | --- | --- | --- | --- | --- | --- | --- | --- |
|  |  | **whole worms** | **heads** | **tails** | **total**^3^ | **whole worms** | **heads** | **tails** | **total**^3^ |  |
| 1 | 2381 | 5 | 1 | 0 | 6 | 0 | 0 | 0 | 0 | 6 |
|  | 2589 | 0 | 4 | 0 | 4 | 0 | 0 | 0 | 0 | 4 |
|  | 2833 | 7 | 8 | 0 | 15 | 0 | 0 | 0 | 0 | 15 |
|  | 2853 | 5 | 11 | 2 | 16 | 0 | 0 | 0 | 0 | 16 |
|  | 2959 | 6 | 9 | 1 | 15 | 0 | 0 | 0 | 0 | 15 |
|  | 3124 | 21 | 9 | 0 | 30 | 0 | 0 | 0 | 0 | 30 |
|  | 3262 | 9 | 5 | 0 | 14 | 0 | 0 | 0 | 0 | 14 |
|  | 3292 | 23 | 19 | 5 | 42 | 1 | 1 | 1 | 2 | 44 |
| 2 | 0752 | 0 | 0 | 0 | 0 | 0 | 0 | 0 | 0 | 0 |
|  | 2445 | 0 | 0 | 0 | 0 | 0 | 0 | 0 | 0 | 0 |
|  | 2488 | 0 | 0 | 0 | 0 | 0 | 0 | 0 | 0 | 0 |
|  | 2975 | 0 | 0 | 0 | 0 | 0 | 0 | 0 | 0 | 0 |
|  | 3069 | 0 | 0 | 0 | 0 | 0 | 0 | 0 | 0 | 0 |
|  | 3188 | 0 | 0 | 0 | 0 | 0 | 0 | 0 | 0 | 0 |
|  | 3243 | 0 | 0 | 0 | 0 | 0 | 0 | 0 | 0 | 0 |
|  | 3253 | 0 | 0 | 0 | 0 | 0 | 0 | 0 | 0 | 0 |
| 3 | 1298 | 0 | 0 | 0 | 0 | 0 | 0 | 0 | 0 | 0 |
|  | 1353 | 0 | 0 | 0 | 0 | 0 | 0 | 0 | 0 | 0 |
|  | 2886 | 0 | 0 | 0 | 0 | 0 | 0 | 0 | 0 | 0 |
|  | 3343 | 0 | 0 | 0 | 0 | 0 | 0 | 0 | 0 | 0 |
|  | 3881 | 1 | 0 | 0 | 1 | 0 | 0 | 0 | 0 | 1 |
|  | 7440 | 0 | 0 | 0 | 0 | 0 | 0 | 0 | 0 | 0 |
|  | 7491 | 0 | 0 | 0 | 0 | 0 | 0 | 0 | 0 | 0 |
|  | 7992 | 0 | 0 | 0 | 0 | 0 | 0 | 0 | 0 | 0 |
| ^1^ Treatment Group 1 = untreated control  2 = Advocate® Spot-on at 0.1 mL/kg on SDs -10 and 18  3 = Advocate® Spot-on at 0.1 mL/kg on SDs 53, 81 and 109  ^2^ Viable plus dead nematodes  ^3^ Whole nematodes plus number of heads or tails | | | | | | | | | | |

| **Additional file 1: Table S3.** *A. abstrusus-*counts at necropsy (study no. 3) | | | | | | | |
| --- | --- | --- | --- | --- | --- | --- | --- |
| **Study group**^1^ | **cat ID** | ***A. abstrusus*** | | | ***T. brevior*** | **living larvae**^2^ | **faecal examination (LPG)**** |
|  |  | **viable** | **non viable** | **total** | **total** |  |  |
| 1 | 5273 | 86 | 2 | 88 | 2 | +++ | xxx |
|  | 3892 | 41 | 4 | 45 | 4 | +++ | xxx |
|  | 0524 | 21 | 0 | 21 | 4 | 0 | x |
|  | 1084 | 3 | 0 | 3 | 4 | 0 | xx |
|  | 2801 | 25 | 5 | 30 | 4 | +++ | xxxx |
|  | 1985 | 56 | 2 | 58 | 0 | +++ | xxxx |
|  | 8044 | 73 | 4 | 77 | 1 | +++ | xxxx |
|  | 6681 | 45 | 0 | 45 | 3 | + | xx |
| 2 | 7690 | 0 | 0 | 0 | 0 | 0 | 0 |
|  | 0642 | 0 | 0 | 0 | 0 | 0 | 0 |
|  | 1916 | 0 | 0 | 0 | 0 | 0 | 0 |
|  | 3834 | 0 | 0 | 0 | 1 | 0 | 0 |
|  | 2622 | 0 | 0 | 0 | 0 | 0 | 0 |
|  | 9234 | 0 | 0 | 0 | 0 | 0 | 0 |
|  | 3708 | 0 | 0 | 0 | 0 | 0 | 0 |
|  | 6582 | 0 | 0 | 0 | 0 | 0 | 0 |
| 3 | 0509 | 0 | 1 | 1 | 0 | 0 | 0 |
|  | 9128 | 0 | 0 | 0 | 0 | 0 | 0 |
|  | 0859 | 0 | 0 | 0 | 0 | 0 | 0 |
|  | 1201 | 0 | 0 | 0 | 0 | 0 | 0 |
|  | 5170 | 0 | 0 | 0 | 0 | 0 | 0 |
|  | 6421 | 0 | 0 | 0 | 0 | 0 | 0 |
|  | 6407 | 0 | 0 | 0 | 0 | 0 | 0 |
|  | 9089 | 0 | 0 | 0 | 0 | 0 | 0 |
| 4 | 3728 | 0 | 0 | 0 | 2 | 0 | 0 |
|  | 1361 | 0 | 0 | 0 | 0 | 0* | 0 |
|  | 4648 | 0 | 0 | 0 | 0 | 0 | 0 |
|  | 4646 | 0 | 0 | 0 | 0 | 0 | 0 |
|  | 4593 | 0 | 0 | 0 | 0 | 0 | 0 |
|  | 8516 | 0 | 0 | 0 | 0 | 0 | 0 |
|  | 2793 | 0 | 0 | 0 | 0 | 0 | 0 |
|  | 3922 | 0 | 0 | 0 | 0 | 0 | 0 |
| ^1^ Treatment Groups  1 = Placebo treated control group SDs -4 and 24  2= Advocate® Spot-on at 0.1 mL/kg on SDs -4 and 24  3 = Advocate® Spot-on at 0.1 mL/kg on SDs -10 and 18  4 = Advocate® Spot-on at 0.1 mL/kg on SD -20 and 8  * 1 dead larva  ^2^living larvae: + small amount, ++ medium amount, +++ large amount  ** Results of faecal examination of samples collected during necropsy: x 1-20 larvae, xx 20-100 larvae, xxx 100-200 larvae, xxxx >200 larvae | | | | | | | |
